# The impact of stress management training on stress-related coping strategies and self-efficacy in hemodialysis patients: a randomized controlled clinical trial

**DOI:** 10.1186/s40359-021-00678-4

**Published:** 2021-11-10

**Authors:** Zohreh Ghasemi Bahraseman, Parvin Mangolian Shahrbabaki, Esmat Nouhi

**Affiliations:** 1 Nursing Research Center, Razi Faculty of Kerman University of Medical Sciences, Kerman, Iran; 2 Department of critical Care Nursing, Razi Faculty of Nursing and Midwifery, Kerman University of Medical Sciences, Kerman, Iran; 3 Department of Medical Surgical Nursing, Razi Faculty of Nursing and Midwifery, Kerman University of Medical Sciences, Haft-Bagh Highway, PO Box 7716913555, Kerman, Iran

**Keywords:** Stress management training, Stress coping strategies, Self-efficacy, Hemodialysis

## Abstract

**Introduction:**

Dialysis causes many psychological and social problems, such as stress and inefficiency in patients, which should be considered in health promotion interventions. Therefore, this study aimed to determine the impact of stress management training on stress coping strategies and the self-efficacy of these patients in southeastern Iran.

**Methods:**

This quasi-experimental study was a randomized controlled clinical trial was conducted on hemodialysis patients from December 2019 to January 2020. Patients who met inclusion criteria were selected with the convenience sampling method and divided into the two groups of intervention (n = 30) and control (n = 30) by using the block randomization method. Participants in the intervention group were trained in a stress management training program in 8 one-and-a-half-hour sessions, held twice a week. Data were measured with stress coping strategies and general self-efficacy questionnaires before, immediately, and 1 month after the intervention. SPSS18 was used for data analysis.

**Results:**

The study results showed that the scores of stress coping strategies and the self-efficacy score in all their dimensions were significantly different between the intervention and control groups (*P* < 0.001).

**Conclusion:**

The present study results showed that stress management training programs promoted stress coping strategies and self-efficacy in hemodialysis patients. Health planners and nurses are recommended to use these easy, feasible, and inexpensive interventions to reduce stress and increase self-efficacy.

*Trial registration* Iranian Registry of Clinical Trials (IRCT): IRCT20160914029817N10. Date of registration: October 7, 2021. URL: https://en.irct.ir/trial/58540. Registration timing: a retrospective.

## Introduction

Chronic kidney disease is the gradual loss of kidney function over time. The primary treatment for end-stage renal disease is dialysis and kidney transplantation [1]. In most countries, the incidence of this disease is more than 200 individuals per million people a year [2]. Hemodialysis is the most common treatment for patients with end-stage renal disease [3]. Dialysis is a stressful process with many psychological and social problems that can lead to mental disorders in patients [4]. In addition to chronic disease, these patients face many stressors, including problems related to treatment, pain, feeling of restlessness, food and fluid limitations, fatigue, weakness, stress, and feeling of inefficiency [5]. Rapid changes in the physical and mental conditions of patients undergoing hemodialysis put them at serious risks [6]. Paying attention to the mental stressors of these patients is very important because they lead to reduced adherence to therapeutic regimens and increased mortality and admission [7]. The adverse effects of stress can put pressure on people and have physical, behavioral, and psychological consequences such as anxiety, worry, mood swings, and physical illnesses. Therefore, recognition of stress and its factors is essential [8].

In Iran, the number of patients undergoing dialysis is estimated at 30,800, of which 29,200, or about 95%, are undergoing hemodialysis [9]. According to available statistics, there is a 16% annual increase in patients undergoing hemodialysis in Iran. Because home care nursing has not made significant progress, patients' psychological problems are not commonly addressed [10].

Stress management is a helpful skill in people. Stress coping skills prepare individuals to better cope with life's needs and challenges [11]. Stress coping skills are behavioral-cognitive activities and processes to prevent, manage, and reduce stress. Coping with stress and the type of stress response is more important than the nature of stress [12]. According to studies, stress management improves mental health and performance because it activates cognition and beliefs. Preventive strategies usually involve trying to achieve various stress management techniques, so people can learn to manage stress before it causes psychological and physical problems [13]. El-Monshed's study showed that cognitive-behavioral nursing interventions reduce the rate of depression and anxiety in patients undergoing maintenance hemodialysis [14]. El-rtreby's study showed that self-management programs positively affect the deterioration of quality of life in patients with chronic kidney disease [15]. Nohi's study showed that problem-based coping strategies are associated with reduced perceived stress and improved quality of life in coronary heart patients [16]. One of the most effective ways to achieve this result is stress management training, which is a type of cognitive-behavioral therapy. Stress management group training is an accurate, multidimensional therapeutic intervention that does not aim to eliminate stress completely but encourages clients to consider stressors as a threat and solve them [17]. The SMT program increases the individual's awareness of the subtle stress response processes through cognitive restructuring, coping skills training, and social support [18].

Self-efficacy is the belief one has in executing a specific task successfully to obtain a certain outcome. Bandura et al. believe that self-efficacy is strengthened in individuals when facing challenges with sequential behaviors [19]. Self-efficacy is one’s sense of mastery over certain activities. It is a person's judgment of their ability to perform a particular activity; thus, self-efficacy plays an important role in regulating emotional states [20]. Self-efficacy is a psychological concept that focuses on the individual's perception of his skills and abilities to successfully deliver a good performance [21]. People with strong self-efficacy are diligent and perseverant in performing tasks compared with those with poor self-efficacy. People with strong self-efficacy believe that they can increase their ability to change environmental challenges, while people with poor Self-efficacy generally believe that they cannot act in a significant way [22]. Among people's true beliefs, self-efficacy plays a significant role in facing obstacles, failures, and setbacks. Belief in self-efficacy is one of the important and influential factors in changing health-related behaviors [22, 23].

In general, with the high prevalence of chronic kidney disease and a significant increase in the number of hemodialysis patients, the need to perform hemodialysis lasting 2 to 4 h twice a week and being on the waiting list for a kidney transplant can cause stress and inefficiency in these patients. Therefore, it is necessary to plan and implement interventions to improve stress and increase self-efficacy in these patients. A review of the literature showed limited studies on stress management training in such patients. Therefore, this study aimed to determine the impact of stress management training on stress coping strategies and the self-efficacy of these patients in southeastern Iran**.**

## Methods

### Design and participants

This quasi-experimental study was randomized controlled clinical trial, with allocation ratio of 1:1. was conducted in the hemodialysis department of a hospital affiliated with Jiroft University of Medical Sciences. The reason for choosing this center was the number of and ease of access to patients. This study lasted from December 2019 to January 2020.

#### Inclusion criteria

1. Physical and mental ability to complete the questionnaire.
2. Ability to understand the Persian language.
3. Absence of a stressful event at least 6 months before the study.
4. Not participating in stress management workshops.
5. Being between 17 and 65 years old.
6. At least 3 months have passed since the beginning of their dialysis.
7. No substance abuse or mental illness that requires medication.

#### Exclusion criteria

1. Not participating in training sessions (two sessions).
2. Occurrence of a stressful and severe event during the study.

### Sample size and sampling

All 85 patients in the dialysis center were purposefully evaluated in terms of inclusion criteria. Inclusion criteria included patients aged 17–65 years with physical and mental ability to complete the questionnaire and understand Persian, with no stressful experience at least 6 months before the study, no participation in stress management workshops, the passage of at least 3 months since the beginning of dialysis, and no substance abuse or mental illness that required medication. Exclusion criteria included absence from training sessions (two sessions) and a stressful and severe event during the study.

### Randomization

**Fig. 1:**
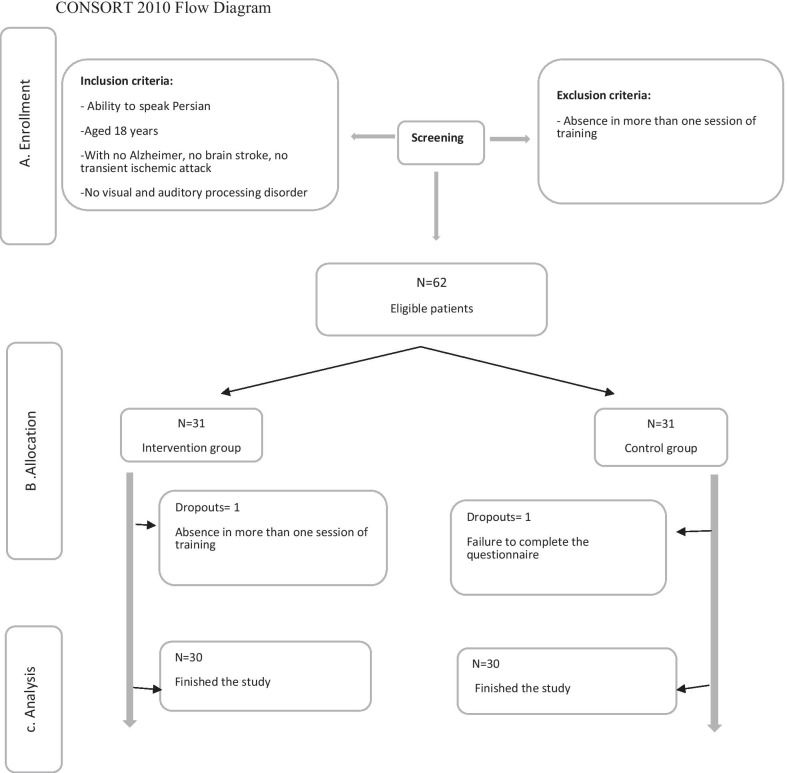
Explanation of sample size and sampling

Out of 85 patients, 65 eligible patients were divided into intervention and control groups with block randomization method. Five samples were excluded from the study for various reasons, including the use of sedatives and the death of first-degree relatives, so the results of 60 samples were analyzed. According to CONSORT 2010 Flow Diagram (Fig. 1).

### Data collection

#### Instrument

A three-part questionnaire was used to collect demographic and background information form, the Billings and Moss coping strategy scale (CSS), and Sherer general self-efficacy scale (GSES). The tools used in this study were translated into Persian, and the language of the used instruments was Persian.

Demographic information included age, sex, level of education, underlying illness, history of mental disorders, use of psychiatric drugs, history of participating in stress management programs, the experience of a severely stressful event during the previous 6 months, disease status, whether fistulas were used for dialysis or catheters, how many times they were hemodialyzed weekly, and how long they had been hemodialyzed.

The coping strategy scale was developed by Billings and Moss (1982). This scale has 32 questions and measures five areas of coping strategies: emotion-focused coping (11 questions), problem-focused coping (3 questions), coping based on the evaluation of the situation (5 questions), coping based on achieving social support (4 questions), and coping based on physical inhibition or on the somatization of problems (9 questions). The scale is scored from zero (never) to three (always). The minimum score is 0, and the maximum is 96. Scores 0–32 show that coping strategy use is low. Scores 32–48 show that coping strategies use is moderate. Scores above 48 show that coping strategy use is high. The internal consistency reliability coefficient was 0.78, and the content validity was verified by experts [24].

Sherer et al. (1982) developed the general self-efficacy scale to measure people’s beliefs in their capabilities to overcome different situations. Seventeen items measure general self-efficacy, with three educational, professional, and social dimensions rated on a five-point scale. Items 1, 3, 8, 9, 13, 15 increase their scores from right to left from strongly disagree to strongly agree (1: strongly disagree, 2: disagree, 3: relatively agree, 4: agree, and 5: strongly agree). The scores of other items increase reversely from left to right (5: strongly disagree, 4: disagree, 3: relatively agree, 2: agree, and 1: strongly agree). The total score is between 17 and 85. The scores 17–34 show poor levels of SE, the scores 34–51 show moderate levels of SE, and scores above 51 show very high levels of SE. Sherer reported the internal consistency and reliability coefficients as 0.76, and the validity of this scale was obtained through construct validity [25].

### Intervention

To conduct the study, the researcher referred to the dialysis ward of Imam Khomeini hospital in Jiroft and obtained the necessary permits. After selecting eligible patients, she allocated participants into intervention and control groups (*n* = 60) using the convenience sampling method. Then, informed written consent was obtained from the patients, and the necessary information about the study objectives was provided to them. The patients of both groups completed the tools. Then, the patients in the intervention group and were trained in eight one-and-a-half-hour sessions twice a week in addition to their routine dialysis treatment. The training sessions included group discussions, questions and answers, and homework and exercises to be done at home. The training sessions were as follows (20) (Table 1). The patients and trainer were not blind to group tasks. However, outcome assessor did not know the purpose and hypothesis of the study, and was blind to when the statistical analysis had been completed.

**Table 1:** The content of the stress management program

Sessions	Summary of the sessions
Session 1	Introducing and getting to know the group members, explaining the group rules and norms, introducing the stress management program
Session 2	Introducing the importance and necessity of stress management skills training, providing definitions of stress, addressing the differences between people in the face of stress and the cause of differences
Session 3	Introducing the general effects of stress on different organs of the body and evaluating participants' behavioral, psychological and physical aspects in the face of stress
Session 4	Examining the coping styles of people in stressful situations, providing mental and intellectual cooperation to cope with stress, introducing problem-focused and emotion-focused methods as coping strategies with stress
Session 5	Introducing the first step of stress coping skills, focusing participants on being aware of their emotions, learning study skills and time management
Session 6	Strengthening self-confidence, self-esteem, coping with anxiety and inefficiency
Session 7	Addressing the second step of stress management skills and teaching long-term and short-term methods
Session 8	Reviewing past sessions, preparing participants to complete group sessions, focusing on generalizing the results of sessions to outside the group

The patients in the control group received only routine care during this period. Both groups completed the coping strategy scale and general self-efficacy scale again immediately and 1 month after the intervention (follow-up). The language of the instruments used was translated into fluent Persian. A trained researcher performed the intervention on participants in groups of 5 to 6 during dialysis when their vital signs and physical condition were stable. The sessions were conducted on days when the patients in the control group were not present in the dialysis center to control the effect of training on the control group.

### Data analysis

SPSS18 was used in this study. Descriptive statistics (frequency, percentage, mean and standard deviation) were used to describe the demographic and background characteristics of the research groups and other study variables. Independent *t*-test and Fisher’s exact test were used to compare the demographic and background characteristics of the samples in the intervention and control groups. Independent *t*-test and Mann–Whitney U tests were used to compare the mean scores of stress coping strategies, and repeated measures ANOVA and ANCOVA were used to compare the mean scores of self-efficacy, before and immediately and 1 month after the intervention.

### Ethical considerations

This study was conducted with the code of ethics IR.KMU.REC.1398.325 issued by Kerman University of Medical Sciences and after acquiring permission from Razi School of Nursing and Midwifery and the hospital management. The participants were informed about the voluntary entrance to and withdrawal from the study and the study objectives and application of results. The results of the study were also given to the authorities if necessary. This research was conducted based on the religious, legal, and professional principles of society. the study was registered retrospectively: IRCT20160914029817N10. Date of registration: October, 7, 2021.

## Results

The mean ages of the samples in the intervention and control groups were 46.00 ± 2.00 and 49.37 ± 1.91. No significant difference was found between the two groups of intervention and control in age, sex, level of education, history of mental disorders, being on the waiting list for the transplant, underlying disease, experience of a severely stressful event during the previous 6 months, history of participating in stress management programs, and history of using psychiatric drugs (Table 2).

**Table 2:** Comparison of demographic information score of patients undergoing hemodialysis in Imam Khomeini hospital affiliated to Jiroft University of Medical Sciences

Variable	Group					
	Intervention		Control		Independent t-test	*P* value
	Mean	SD	Mean	SD		
Age	46.00	2.00	49.37	1.91	− 1.22	0.23
	n	%	n	%		
Sex						
Female	17	56.7	13	43.3	χ^2^ = 1.07	0.30
Male	13	43.3	17	56.7		
Education						
Uneducated	12	40	20	66.7	χ^2^ = 4.49	0.11
Diploma	7	23.3	3	10		
Associate degree and higher	11	36.7	7	23.3		
History of mental disorders						
Yes	4	13.3	0	0	Fisher's exact test = 4.29	0.11
No	26	86.7	30	100		
Are you in the waiting list for a kidney transplant?						
Yes	23	76.7	23	76.7	–	–
No	7	23.3	7	23.3		
Do you have any other underlying diseases?						
Yes	29	96.7	29	96.7	–	–
No	1	3.3	1	3.3		
Experience of a stressful event in the last 6 months						
Yes	0	0	0	0	–	–
No	30	100	30	100		
History of participation in stress management programs						
Yes	0	0	0	0	–	–
No	30	100	30	100		
History of using psychiatric drugs						
Yes	3	10	0	0	Fisher's exact test = 3.16	0.24
No	27	90	30	100		

The results of the study showed that the score of stress coping strategies was significantly different between the two groups in dimensions of coping with cognitive evaluation, problem-solving, excitement, social support, and control of physical problems; in other words, the scores of stress coping strategies in the intervention group in all of the dimensions were higher than those of the control group during the study (*P* < 0.001). At the same time, the treatment group improved compared to the initial state (Table 3).

**Table 3:** Comparison of mean score of stress coping strategies in patients undergoing hemodialysis before, immediately and 1 month after intervention

Variable	Group					
	Intervention		Control		Statistical analysis	*P* value
	Mean	SD	Mean	SD		
Before intervention	30.93	5.37	31.60	6.65	− 0.43*	0.67
Immediately after intervention	39.87	3.93	30.17	6.23	− 5.54**	< 0.001
One month after intervention	40.73	4.36	31.00	7.30	− 5.01**	< 0.001
Repeated measures ANOVA	102.14		1.81			
*P* value	< 0.001		0.18			

*t Independent t-test

**Z Mann–Whitney U test

The results of the study showed that the score of self-efficacy was significantly different between the two groups before the intervention in educational, professional, social dimensions; in other words, the scores of self-efficacy in the intervention group in all of the dimensions were higher than those of the control group during the study (*P* < 0.001). The self-efficacy score was higher in the intervention group than in the control group at the beginning of the study. Therefore, to control the effect of the confounding variable, the pre-intervention score was entered as a confounding variable in the analysis variance model in repeated measurment. The results showed only that variable of the group was the cause of differences in the score of self-efficacy. In contrast, the treatment group improved compared to the initial state (Table 4).

**Table 4:** Comparison of mean self-efficacy score in hemodialysis patients before, immediately and 1 month after intervention

Variable	Group					
	Intervention		Control		Mean difference	*P* value*
	Mean	SD	Mean	SD		
Before intervention	52.53	5.84	49.77	4.40	2.77	0.04
Immediately after intervention	65.30	5.15	50.00	4.94	13.78	< 0.001
One month after intervention	65.23	5.31	49.50	4.92	14.28	< 0.001

Source of difference	Sum of squares	df	F	*P* value	Eta2
Time	0.25	1	0.05	0.82	0.001
Pre-intervention score	0.43	1	0.09	0.76	0.002
Group* time interaction	1.74	1	0.38	0.54	0.007
Group	5496.67	1	170.32	< 0.001	0.75
Error	1839.5	57			

F = repeated measures ANOVA

*Bonferroni: adjustment for multiple comparisons

## Discussion

This study aimed to investigate the effect of stress management training on stress coping strategies and self-efficacy in hemodialysis patients, and the results of the present study demonstrated the effect of this training method on the scores of stress coping strategies and self-efficacy. According to the present study results, the mean score of stress coping strategies in the intervention group increased immediately and 1 month after the intervention. Poorgholami et al. reported that the level of stress in patients of the intervention group significantly decreased after the intervention compared with the control group [26]. Stächele et al. found that an internet-based short-term stress management program improved stress coping skills, sleep quality, and well-being and reduced the perceived stress of the staff [27]. Alkhawaldeh et al. showed a significant difference between intervention and control groups in both levels of job stress and coping strategies [28]. Ertekin Pinar et al. found a reduction in the mean depression score of both groups during the study, but this decrease was greater in the intervention group. In the post-training evaluation, the mean stress score in the experimental group was lower than that of the control group, and the intervention group had higher scores in self-confidence and social support, which are subscales of stress coping strategies [29]. Ata et al. also stated that stress management programs reduced stress indicators, risk of mental disorder, and the caregiver’s burden and increased problem-solving and emotion-focused skills in the study group [30]. Oztürk et al. showed that the mean score of stress coping styles was significantly different between the intervention and control groups after the intervention [31]. Nazer et al. showed that the intervention group's mean pre- and post-test stress scores differed significantly, showing reduced stress in the intervention group [32]. All of these studies supported the results of the present study. It is inferred that coping patterns are important because they facilitate handling a stressful experience. If someone is going through a tough time, positive coping patterns provide extra resources to help that person deal with the demands caused by the stressor.

According to the results of the present study and previous studies, a stress management training program can effectively improve stress coping strategies and reduce stress in patients and individuals. Patients undergoing hemodialysis experience stress in their lives due to factors such as physical dependence on devices, limitations in physical function, changes in sexual function and diet, fluid restriction, consumption of large amounts of drugs for treatment, and loss of appetite and energy [33], so it seems necessary to use an effective intervention in order to reduce stress and help them cope with it. Today, it has been recognized that people respond differently to stressful situations. Using different methods to cope with stress has different consequences on individuals’ physical and mental health. Coping skills are passive or active efforts used in response to threatening situations, and they help patients reduce emotional distress [34]. Dealing with chronic illness is always a challenging and threatening process, and if coping strategies are used effectively, they can help improve performance and well-being [35]. Stress management training is one of the most effective methods, and it is an accurate, multidimensional, and therapeutic intervention. The purpose of this intervention is not to completely eliminate stress but to encourage clients to consider stressful situations as threats and solve them [36]; this method can be used for chronic kidney patients. It seems that stress coping strategies can help patients' minds and bodies adapt. Without it, their bodies might always be on high alert. Over time, chronic stress can lead to serious health problems. Patients should not wait until stress damages their health, relationships, or quality of life.

The present study results showed that the mean score of self-efficacy in the intervention group increased immediately and 1 month after the intervention, and it was significantly higher than that of the control group. The researchers showed that stress management counseling and training could improve and increase self-efficacy [20, 37, 38]. Terp et al. showed that students in the intervention group were better able to manage stress, self-efficacy and self-esteem than those in the control group [36]. Moharrami et al. also showed a significant difference in the mean scores of job stress and SE between the intervention and control groups after the intervention [20]. Jajormaneh et al. argued that the self-efficacy score in the intervention group was significantly higher than that of the control group immediately and 1 month after the intervention [21]. These results suggest that encouraging flexibility in coping strategies would help to improve patients’ self-efficacy.

According to the results of the present study and other studies, a stress management training program can be an effective intervention to promote self-efficacy in individuals. Evidence suggests that increasing the self-efficacy of dialysis patients improves weight control between dialysis sessions, reduces admission, reduces amputation, and improves the quality of life, especially in dialysis patients with diabetes. In addition, increasing self-efficacy leads to behavioral changes, acceptance of treatment, and promotion of physical and mental health [20]. Because the results of studies have shown poor self-efficacy in dialysis patients [20, 39], appropriate methods to improve self-efficacy in these patients are vital. According to the studies and the positive effect of stress management training, it seems that this training method can be useful and effective in improving the self-efficacy of the patients undergoing hemodialysis.

Fatigue and long duration of hemodialysis were among the limitations of the study and patients were reluctant to attend training classes. We tried to motivate patients to participate in classes and hold sessions at times that did not interfere with patients' activities and treatment. Questionnaires were also completed by patients before hemodialysis.

## Conclusion

The present study results showed that the stress management training program promoted stress coping strategies and self-efficacy in hemodialysis patients. Given these results and the importance of stress and poor self-efficacy in these patients, it is recommended that the healthcare staff and nurses use this easy, feasible, and inexpensive intervention while providing healthcare for dialysis patients. Furthermore, managers and nursing education authorities can use this educational method to reduce stress and improve the self-efficacy of these patients.

## Limitations

Fatigue and long duration of hemodialysis were among the limitations of the study and patients were reluctant to attend training classes. We tried to motivate patients to participate in classes and hold sessions at times that did not interfere with patients' activities and treatment. Questionnaires were also completed by patients before hemodialysis.

## Data Availability

The datasets used and/or analyzed during the current study available from the corresponding author on reasonable request.

## Abbreviations

SE: Self-efficacy
GSES: General self-efficacy scale
CSS: Coping strategy scale
CS: Coping strategy
